# Mental Health Outcome Trends in a Nationally Representative Sample of Canadian Migrant Adolescents From 2014 to 2022

**DOI:** 10.1111/josh.70177

**Published:** 2026-06-12

**Authors:** Daniel Ji, Mauricio Coronel‐Villalobos, Monica Rana, Nour Hammami, Elizabeth Saewyc

**Affiliations:** ^1^ University of Regina Faculty of Social Work Regina Saskatchewan Canada; ^2^ Stigma and Resilience Among Vulnerable Youth Centre The University of British Columbia Vancouver British Columbia Canada; ^3^ Child and Youth Studies Trent University Peterborough Ontario Canada

**Keywords:** adolescent, longitudinal, mental health, migrant, trends, well‐being

## Abstract

**Background:**

Migrant youth are at disproportionate risk of mental health challenges. Overcoming barriers to accessing services requires large‐scale data to inform policies and interventions. This study maps mental health outcome trends of migrant youth over 8 years.

**Methods:**

Canadian Health Behavior in School‐aged Children study data from 2014, 2018, and 2022 were analyzed for mental health outcome trends. Age‐adjusted logistic regressions examined health across years stratified by migrant status, separately by sex. Using nonmigrants as the referent group and 2014 as the referent year, contrasts for disparities were examined across migrant status to test widening, narrowing, or stability in differences of outcome prevalences between migrant and nonmigrant youth.

**Results:**

Health worsened from 2014 to 2022 among migrants, especially migrant girls. Compared to 2014, life satisfaction, health, and self‐confidence for migrant youth dropped in 2022. Health complaints and feeling sad/hopeless increased in 2022 among girls. Migrant youth reported fewer health complaints than nonmigrants in 2018 and 2022.

**Implications:**

Investment in free/affordable, culturally safe/relevant, confidential school‐based mental health supports with avenues for community collaboration are recommended.

**Conclusions:**

Mental health outcomes worsened from 2014 to 2022, especially among migrants and girls; however, migrant youth exhibit resilience to adversity.

Migrant adolescents are at higher risk of mental health challenges than nonmigrant adolescents [1, 2]. Evidence suggests that migrant adolescents are at increased risk for mental health problems compared to their nonmigrant peers including internalizing problems, depressive and anxiety symptoms [3, 4], suicidal ideation and suicide attempts [5], depression [2], and posttraumatic stress disorder which can commonly appear in the form of health complaints like headaches, irritability, fatigue, and difficulties sleeping [6]. Nonrefugee migrant youth have been found to report high levels of post‐traumatic stress symptoms and are at risk of developing mental health problems [7]. The disparity in mental health between migrant youth and their native‐born counterparts is a growing public health concern [8]. Of particular concern is a mounting body of evidence indicating that girls are more likely than boys to experience poor mental health outcomes [9], and that more recent cohorts of young people are reporting poorer mental health outcomes than previous generations [9, 10].

One possible explanation for the disparity in mental health outcomes between migrant and nonmigrant youth is the barriers migrants face when trying to access mental health services. Migrant youth have been found to experience stigma surrounding mental illness, fear of being ignored or misunderstood by service providers, confidentiality concerns, mistrust discussing mental health with professionals, mental health illiteracy among family members, lack of culturally appropriate services, and financial costs which could preclude access [11, 12]. Another reason migrant youth may experience poorer mental health outcomes than nonmigrant youth is the prevalence of stressors they experience. According to Berry [13], immigrants may experience stressors associated with the acculturation strategies they use when trying to balance maintenance of heritage culture and adapting to a host culture. Challenges associated with acculturation have been found to contribute to mental health outcomes among migrant youth [14, 15, 16, 17]. Finnigan et al. [11] found that migrant youth in Canada experience stressors related to family and peers, feeling overburdened, isolation, communication difficulties, anxiety, and difficulties at school. Consequently, migrant youth in Canada have been found to be less likely than their Canadian‐born peers to utilize mental health services [18], and immigrant youth in Canada have been found to be more likely to present with a first mental health crisis to emergency departments than their nonmigrant counterparts [19]. A sub‐group of girls who present a particular pattern are migrant girls. They are found to report poorer mental health and depression more frequently compared with their nonmigrant, girl peers in Canada and the United States [20, 21]. This could be understood given the bicultural life that they engage in; while practices at home still largely reflect the original country they migration from, while the girls are acclimatizing to a new culture at school. The culture at school reflects different lifestyles and values, but also new gender‐norms that may challenge migrant girls' perception of identity and roles within the household. Hence, female migrants navigate a dichotomous environment where they largely “role‐play” depending on the setting that they are in. For youth at a sensitive time of their development and identity formation, the divide is bound to result in inner conflict and poor mental health [22, 23, 24, 25].

Even though past studies of mental health outcomes suggest a worsening trend over time for adolescents, especially girls [26, 27, 28], few studies have examined trends in mental health outcomes specifically among migrant youth in Canada. Hammami et al. [28] explain that mental health issues among girls in Canada have worsened due to lower socioeconomic positions and faring worse on indicators of health at several cycles compared to males and more affluent adolescents. They also explain that gender differences and widening inequalities in daily physical activity as well as low life satisfaction stemming from earlier maturation physically, greater stress, and perceived social pressure among girls may contribute to worsening mental health with each successive cycle.

Migration flows changed in Canada between 2014 and 2022, with immigrants making up the largest share of Canada's population as of the 2021 Canadian census [29]. According to Statistics Canada, the Canadian government made a commitment to bring more Syrian refugees into the country. From 2001 to 2010, Afghanistan, Iraq, and Colombia were the main countries of birth of refugees settling in Canada. From 2016 to 2021, however, 60,795 new Syrian‐born refugees were living in Canada, accounting for over one‐fourth of new refugees. Data from Statistics Canada from 2021 indicates that most newcomers to Canada come from India (18.6%), the Philippines (11.4%), and China (8.9%). Nearly 70% of recent immigrants did not report English or French as their first language. Newcomers from conflict situations arriving in lower socioeconomic positions with limited language fluency from 2014 to 2022 may face greater socio‐cultural challenges.

To our knowledge, the only study examining health trends over time among Canadian youth examined socioeconomic inequalities in physical and mental health and wellbeing from 2002 to 2018 [28], with life satisfaction and overall health examined as part of their analysis. To better target interventions for migrant youth well‐being in Canada, research is needed that documents trends in outcomes over time using large‐scale population‐level data. One example of such population‐level data is the Health Behavior in School‐aged Children (HBSC) study. Founded in 1982, the HBSC is a World Health Organization collaborative cross‐national study of adolescent health and well‐being [9]. Since its founding, the Health Behavior in School‐aged Children study collects data on social determinants of health approximately every 4 years and is currently composed of 450 researchers from 51 member countries.

This study was guided by the overarching question: What are the trends in mental health outcomes of migrant adolescents in Canada from 2014 to 2022? Flowing from this question, we ask:

1. What are the changes in mental health prevalence outcomes in migrant and nonmigrant boys and girls in Canada from 2014 to 2022?
2. Are there disparities in Canadian migrant adolescents' mental health outcomes compared to their nonmigrant counterparts in 2014, 2018, and 2022?
3. Are any gaps between migrant and nonmigrant boys' and girls' mental health outcomes narrowing, widening, or unchanged over time?

## 1 Methods

### 1.1 Participants

The total sample of 76,856 participants was comprised of 51.6% girls and 48.4% boys. Migrant youth comprised 14.9% of the overall sample; 19.7% in 2014, 12.1% in 2018, and 11.7% in 2022. Independent samples *t*‐tests indicated that migrant participants were older than their nonmigrant peers (*p* < 0.05). We then examined age differences between migrant and nonmigrant youth by year. There were no significant age differences in 2014 and 2022, but migrant youth were significantly older in 2018. Prevalence estimates by wave disaggregated by sex and migrant status are shown in Table 1.

**TABLE 1:** Demographic information by year.

	2014 (*n =* 29,622)		2018 (*n =* 21,079)		2022 (*n* = 26,155)	
		Mean		Mean		Mean
	*n* (%)	Age (SE)	*n* (%)	Age (SE)	*n* (%)	Age (SE)
Girls	Total = 15,103		Total = 11,075		Total = 13,488	
Migrant	2582 (17.1%)	14.0 (0.1)	1316 (11.9%)	14.2*** (0.1)	1475 (10.9%)	14.1 (0.1)
Nonmigrant	12,520 (82.9%)	14.1 (0.1)	9579 (88.1%)	13.8*** (0.1)	12,012 (89.1%)	13.9 (0.1)
Boys	Total = 14,519		Total = 10,004		Total = 12,668	
Migrant	3245 (22.3%)	14.1 (0.1)	1230 (12.3%)	14.1* (0.1)	1573 (12.4%)	14.1 (0.1)
Nonmigrant	11,275 (77.7%)	14.1 (0.1)	8774 (87.7%)	13.79 (0.1)	11,095 (87.6%)	13.9 (0.1)

*Note*: Table reports weighted *n*'s and percentages.

Abbreviation: CI, confidence interval.

* *p* < 0.05.

*** *p* < 0.001.

### 1.2 Instrumentation

#### 1.2.1 Positive Mental Health Outcomes

We examined three positive mental health outcomes. Overall health was measured by asking participants “Would you say your health is…?”, and given the response options of “excellent,” “good,” “fair,” or “poor.” We dichotomized the item to focus analysis on those who reported their health as “good” or “excellent.”

Life satisfaction was measured on a 10‐point scale using the Cantril Scale (aka., Cantril's Self‐Anchoring Ladder of Life Satisfaction) [24]. The Cantril scale (CS) is a global measure of life satisfaction that was adapted for adolescents by the Health Behavior in School‐aged Children study and is used extensively nationally and internationally for its performance as a simple self‐anchoring scale for measuring adolescent psychosocial health broadly [30, 31, 32]. Participants were shown a picture of a ladder with “10” indicating “the best possible life for you” and the bottom, or zero, indicating “the worst possible life for you”. Participants were asked to mark “Where on the ladder do you feel you stand at the moment?” Consistent with our goal of examining satisfaction with life as well as previous studies using the measure, we decided to categorize scores of 9 or 10 as representative of high life satisfaction [30, 33, 34]. Evidence supporting use of the CS includes good reliability and convergent validity in studies with samples of Scottish adolescents [35], gauging overall adolescent health with Polish adolescents [36], and moderate concurrent validity with measures of self‐efficacy and depression among refugee youth with symptoms of post‐traumatic stress [37].

Self‐confidence was measured using a five‐point Likert type item wherein participants were asked the extent to which they agreed with the statement “I have confidence in myself” from “strongly disagree” to “strongly agree”. We dichotomized the variable to examine those who responded “agree” or “strongly agree.”

#### 1.2.2 Negative Mental Health Outcomes

Two mental health outcomes were included in our study: health complaints (aka. psychosomatic symptoms) and feeling sad or hopeless. Participants were asked about the frequency of occurrence in the past 6 months of headaches, stomachaches, backaches, feeling low (depressed), irritability or bad temper, feeling nervous, sleep difficulties, and dizziness on a 5‐point Likert‐type scale with response options ranging from 1 = “About every day” to 5 “Rarely or never.” Following prior waves of the HBSC‐SCL as well as researchers who have previously used the measure, we dichotomized multiple health complaints defined as the presence of two or more symptoms more than once a week in the last 6 months [38, 39]. Validity and reliability evidence supporting the use of the HBSC‐SCL has been gathered in the form of good construct, convergent and discriminant validity and adequate test–retest reliability [40, 41].

Feeling sad or hopeless was a dichotomous (yes/no) variable which asked participants, “During the past 12 months, did you ever feel so sad or hopeless almost every day for two weeks or more in a row that you stopped doing some usual activities?”

#### 1.2.3 Predictor

Migrant status was used to compare groups with a binary item asking whether participants were born in Canada. This item was asked in a different way in 2014 than in 2018 and 2022. Participants in 2014 were asked “How many years have you lived in Canada?” with one of the response options being “I was born in Canada”. In 2018 and 2022, participants were asked “In which country were you born?” (Canada/Other country/I don't know). As such, we created a “Born in Canada” variable by dichotomizing responses to whether a participant reported being born in Canada. Those who responded “I don't know” in 2018 and 2022 were treated as missing and excluded from analysis.

#### 1.2.4 Covariate

We included age as a covariate in all analyses because the age of onset for many mental disorders is in adolescence [42, 43].

#### 1.2.5 Demographic Information

Data were collected on participants' numerical age and sex registered at birth. Due to gender differences on mental health outcomes in the literature, we conducted analysis separately for boys and girls. Sex registered at birth was used instead of gender because gender was not introduced until the 2022 cycle of the survey. Participants were excluded from analysis if they did not report their sex registered at birth or if they reported their sex registered at birth as neither male nor female.

### 1.3 Procedure

The Health Behavior in School‐aged Children (HBSC) is a cross‐sectional international study about the well‐being of adolescents within their social context (at home and at school and with important others like family and friends) [37]. Data collection usually occurs once every 4 years from children enrolled in public schools ranging in age from 11 to 15 from participating countries and regions (in Canada, these ages correspond with grades 6 through 10) [31]. We used Canadian HBSC data from 2014, 2018, and 2022 to conduct our analyses. Three levels of consent were needed before students could participate (i.e., school jurisdictions, school principals, then active or passive participant consent).

Data are collected using a systematic, two‐stage cluster sampling strategy using schools as the primary sampling unit to ensure representativeness of the sample by age, sex, and type of school, further details on collection are described elsewhere [42]. Data are weighted such that responses from participants from each Canadian province contribute to national results proportionate to the actual student population within that grade at a national level.

### 1.4 Data Analysis

We conducted a trends analysis on data from the Canadian HBSC study for three consecutive waves corresponding with school years 2014/15, 2018/19, and 2022/23 following Homma et al. [43] Trends analysis uses logistic regression modeling to test for (1) differences in the likelihood of an outcome variable over time compared to a reference year, (2) differences between groups compared to a reference group by year, and (3) whether any differences observed between groups widen, narrow, or remain constant through the use of an interaction term in the regression model (i.e., year by group).

Our trends analysis consisted of three steps: First, we described sample demographic information disaggregated by survey year and migration status. We examined outcome variable prevalence by year (self‐reported overall health, life satisfaction, self‐confidence, health complaints, and feelings of sadness or hopelessness). We then analyzed trends of prevalence of mental health outcomes across years stratified by migration status using age‐adjusted logistic regressions, which were conducted separately for boys and girls. Second, we examined contrasts for gaps across migration status stratified by year using nonmigrant youth as the reference group and 2014 as the reference year.

To examine relative change between the reference year and other years, our third step was to examine whether differences in the prevalence of outcome variables between migrant and nonmigrant youth narrowed, widened, or remained constant for girls and boys from 2014 to 2018 and 2022. To accomplish that, we computed an interaction term (migration status by survey year) in logistic regression models such that the full model for each outcome variable included migration status, survey year, migration status by survey year interaction and age. Nonmigrant youth and 2014 served as the reference groups. A statistically significant interaction term indicates that the gap in recent rates of mental health outcomes between migrant and nonmigrant youth has widened or narrowed over time. The interaction term essentially serves as a ratio of odds ratios (i.e., a ratio of the OR of a mental health outcome by migrant status group for a given year to the odds of the same mental health outcome among migrant versus nonmigrant youth for a reference year).

Interpretation of this ratio of ratios requires attention to both the main effect OR in the model as well as the interaction term. Determination of whether the gap is narrowing or widening is made by using the OR for the migration‐status based difference in a given year in combination with the reference year and the interaction OR [43]. Table 2 provides a reference for instructions on how to interpret odds ratios for gap analysis.

**TABLE 2:** Interpretation of odds ratios for gap analysis.

Year	Original ORs	ORs for interaction terms	Outcome variable
2018, 2022	> 1	> 1	Widening
2018, 2022		< 1	Narrowing
2014 (reference)			
2018, 2022	< 1	> 1	Narrowing
2018, 2022		< 1	Widening
2014 (reference)			

We analyzed Canadian HBSC data using Complex Samples in SPSS (v. 29) to account for the weighting of the data and the cluster sampling design. Pearson chi‐square tests of outcome variables by predictor were significant in 2014 for health complaints and self‐confidence. The same tests in 2018 were significant for overall health, life satisfaction, and health complaints. In 2022, chi‐square tests by predictor were significant for health complaints and self‐confidence. As such, further trends analysis was warranted.

## 2 Results

Table 3 shows trends in mental health outcomes using 2014 as reference year. Compared to 2014, both migrant girls and migrant boys were less likely to report that they were highly satisfied (9 or 10) with their lives in both 2018 and 2022. Nonmigrant girls and boys were less likely to report that they had high levels of life satisfaction in 2022 compared to 2014. Migrant girls were less likely to report their overall health as good or excellent in both 2018 and 2022 compared to 2014. Nonmigrant girls were less likely to report good or excellent health in 2022 compared to 2014. Both migrant and nonmigrant boys were less likely to report good or excellent overall health in 2022, though migrant boys were less likely than their nonmigrant counterparts to report having good or excellent overall health. All participants were less likely in 2018 and 2022 compared to 2014 to report that they agreed or strongly agreed that they had confidence in themselves. Both migrant and nonmigrant girls were more likely to report multiple health complaints in 2022 compared to 2014. Nonmigrant boys were more likely than migrant boys to report multiple health complaints in 2022 compared to 2014. All girl participants were more likely to report that they felt sad or hopeless in the past 12 months in 2018 and 2022 compared to 2014, though the odds ratios were slightly higher for migrant girls. Nonmigrant boys were more likely to report feeling sad or hopeless in the past 12 months in 2018 compared to 2014.

**TABLE 3:** Trends in mental health outcomes between 2014 and 2022, by migrant status.

	Prevalence comparison			Trend comparison	
				2018^a^	2022^a^
	2014 (%)	2018 (%)	2022 (%)	aOR (95% CI)	aOR (95% CI)
Life satisfaction					
Girls					
Migrant	25.7	19.9	19.9	0.75* (0.59, 0.97)	0.75* (0.58, 0.98)
Nonmigrant	24.7	24.4	17.9	0.92 (0.83, 1.02)	0.62*** (0.56, 0.70)
Boys					
Migrant	30.6	25.0	22.1	0.76* (0.60, 0.97)	0.65*** (0.51, 0.82)
Nonmigrant	31.7	32.6	26.9	0.99 (0.89, 1.09)	0.77*** (0.69, 0.85)
Health					
Girls					
Migrant	81.6	76.8	68.2	0.76* (0.60, 0.96)	0.49*** (0.39, 0.61)
Nonmigrant	81.1	81.8	69.9	0.98 (0.85, 1.13)	0.51*** (0.45, 0.57)
Boys					
Migrant	86.3	83.2	79.7	0.79 (0.61, 1.03)	0.61*** (0.48, 0.78)
Nonmigrant	84.3	85.8	82.0	1.07 (0.93, 1.22)	0.82*** (0.73, 0.92)
Self‐confidence					
Girls					
Migrant	58.1	48.5	48.5	0.70*** (0.57, 0.85)	0.70*** (0.58, 0.84)
Nonmigrant	55.1	50.8	41.4	0.81*** (0.73, 0.89)	0.56*** (0.51, 0.61)
Boys					
Migrant	77.4	67.1	69.2	0.59*** (0.47, 0.73)	0.64*** (0.51, 0.80)
Nonmigrant	76.5	70.9	69.8	0.72*** (0.64, 0.79)	0.69*** (0.62, 0.76)
Health complaints					
Girls					
Migrant	35.7	40.0	51.3	1.16 (0.93, 1.46)	1.91*** (1.54, 2.36)
Nonmigrant	42.8	43.6	59.0	1.10* (1.00, 1.22)	2.08*** (1.88, 2.30)
Boys					
Migrant	21.2	19.5	23.5	0.88 (0.68, 1.14)	1.14 (0.90, 1.46)
Nonmigrant	23.3	24.1	28.1	1.09 (0.97, 1.22)	1.33*** (1.19, 1.49)
Sad/hopeless					
Girls					
Migrant	32.9	41.2	44.0	1.38*** (1.14, 1.68)	1.57*** (1.29, 1.92)
Nonmigrant	35.6	36.7	43.6	1.12* (1.01, 1.24)	1.49*** (1.34, 1.65)
Boys					
Migrant	20.3	21.6	22.1	1.09 (0.86, 1.38)	1.14 (0.91, 1.44)
Nonmigrant	19.2	22.1	20.7	1.22*** (1.09, 1.36)	1.12 (1.00, 1.25)

*Note*: Data are weighted.

Abbreviations: aOR, age‐adjusted odds ratio; CI, confidence interval.

^a^ Referent year is 2014. Cells not included are due to *n* < 5.

* *p* < 0.05.

*** *p* < 0.001.

Table 4 shows odds ratios for outcome variables by year with comparisons by migrant status. Migrant boys were less likely to report high life satisfaction in 2018 compared to their nonmigrant peers. Migrant girls were less likely than nonmigrant girls to report having good or excellent overall health in 2018 compared to nonmigrant girls. Migrant girls were found to be more likely to report agreeing or strongly agreeing that they had confidence in themselves compared to nonmigrant girls. Migrant girls were less likely to report multiple health complaints in 2014, 2018, and 2022 compared to nonmigrant girls. Migrant boys were less likely to report multiple health complaints compared to nonmigrant boys in 2018 and 2022.

**TABLE 4:** Odds ratios and 95% confidence intervals for all outcome variables by year (2014–2022): comparisons by migrant status.

	2014	2018	2022
	aOR^a^ (95% CI)	aOR^a^ (95% CI)	aOR^a^ (95% CI)
Life satisfaction			
Girls			
Migrant^b^	1.04 (0.88, 1.22)	0.86 (0.70, 1.07)	1.27 (0.99, 1.62)
Boys			
Migrant^b^	0.95 (0.82, 1.10)	0.73** (0.58, 0.91)	0.81 (0.65, 1.00)
Health			
Girls			
Migrant^b^	1.03 (0.87, 1.21)	0.80* (0.66, 0.96)	0.98 (0.81, 1.19)
Boys			
Migrant^b^	1.18 (0.98, 1.41)	0.86 (0.69, 1.08)	0.89 (0.72, 1.10)
Self‐confidence			
Girls			
Migrant^b^	1.12 (0.98, 1.28)	0.97 (0.81, 1.16)	1.38*** (1.17, 1.63)
Boys			
Migrant^b^	1.08 (0.91, 1.28)	0.88 (0.75, 1.04)	1.00 (0.82, 1.20)
Health Complaints			
Girls			
Migrant^b^	0.74*** (0.64, 0.86)	0.78** (0.65, 0.93)	0.69*** (0.57, 0.83)
Boys			
Migrant^b^	0.89 (0.76, 1.04)	0.72** (0.57, 0.89)	0.76** (0.62, 0.93)
Sad/hopeless			
Girls			
Migrant^b^	0.90 (0.79, 1.02)	1.10 (0.93, 1.31)	0.95 (0.79, 1.14)
Boys			
Migrant^b^	1.07 (0.91, 1.25)	0.95 (0.78, 1.16)	1.09 (0.88, 1.34)

*Note*: Data are weighted.

Abbreviations: aOR, adjusted odds ratio; CI, confidence interval.

^a^ Odds ratios adjusted for age.

^b^ Born in Canada is the reference group.

* *p* < 0.05.

** *p* < 0.01.

*** *p* < 0.001.

Table 5 shows interaction terms of all mental health outcome variables by migrant status and year. To determine whether gaps between migrant and nonmigrant youth are widening, narrowing, or remaining consistent requires an examination of the original between‐groups odds ratios (Table 3), in combination with between‐group gaps (Table 4) as well as the interaction term (outcome by year) of the full model (Table 5). Even though migrant girls and boys showed lower life satisfaction in 2018 and 2022 compared to 2014, satisfaction between migrant and nonmigrant girls remained consistent across years, and the lack of significant interaction term indicates consistency across waves. Migrant boys self‐reported lower life satisfaction than nonmigrant boys in 2018, which was a drop from 2014; however, the lack of a significant interaction term suggests that the gap was not significantly widening or narrowing over time. The overall health of migrant girls was significantly lower than that of nonmigrant girls in 2018 and dropped from 2014; however, the lack of a significant interaction term suggests that the gap is not significantly narrowing or widening over time. The overall health of migrant (and nonmigrant) boys declined in 2022 compared to 2014, but the difference between migrant and nonmigrant boys was not significant in 2018 or 2022. Even though the interaction term is significant, the fact that the gap between groups is not significant suggests the gap is not widening or narrowing over time. The self‐confidence of all youth declined from 2014 to 2018 and 2022. Based on the differences between migrant and nonmigrant youth observed in Table 4, one of the interaction terms was found to be widening or narrowing. In 2022, self‐confidence among migrant girls was higher than among their nonmigrant counterparts. Based on the significant interaction term (migrant girls × 2022) that is less than one, indicating that the gap is narrowing. Health complaints remained consistently lower among migrant girls in all three waves, and migrant boys had a lower likelihood of multiple recurring health complaints in 2018 and 2022. The fact that the interaction term odds ratios were not significant indicates that this trend is consistent across waves. Girls were more likely to report feeling sad or hopeless in the past year in 2018 and 2022 compared to 2014; however, the gaps between migrant and nonmigrant girls were not significant in any of the waves, nor were any interaction terms significant. This suggests that all girls were feeling more sad or hopeless in 2018 and 2022 compared to 2014, but there were no gaps between migrants and nonmigrants.

**TABLE 5:** Trends in mental health outcomes: interactions by migrant status and year.^a^

	Life satisfaction	Health	Self‐confidence	Health complaints	Sad/hopeless
	aOR^b^ (95% CI)	aOR^b^ (95% CI)	aOR^b^ (95% CI)	aOR^b^ (95% CI)	aOR^b^ (95% CI)
Girls					
Migrant × 2022	0.82 (0.61,1.11)	1.04 (0.81,1.34)	0.79* (0.64,0.98)	1.09 (0.86,1.38)	0.95 (0.76,1.18)
Migrant × 2018	1.21 (0.92,1.60)	1.29 (0.98,1.68)	1.16 (0.93,1.44)	0.95 (0.75,1.20)	0.81 (0.65,1.01)
Boys					
Migrant × 2022	1.19 (0.92,1.55)	1.34* (1.01,1.77)	1.08 (0.84,1.39)	1.16 (0.90,1.50)	0.98 (0.76,1.27)
Migrant × 2018	1.31 (1.0,1.71)	1.35* (1.00,1.82)	1.22 (0.96,1.56)	1.23 (0.94,1.62)	1.12 (0.86,1.45)

*Note*: Data were weighted.

Abbreviations: aOR, adjusted odds ratio; CI, confidence interval.

^a^ Born in Canada and year 2014 are the reference groups.

^b^ Adjusted model included migrant status, survey year, and age along with the migrant status × year interactions.

* *p* < 0.05.

## 3 Discussion

This study mapped trends over time of mental health outcomes in a nationally representative sample of Canadian adolescents from 2014 to 2022. Overall, the mental health outcomes for both migrant and nonmigrant youth worsened in 2022 from 2014. The decline in mental health outcomes was more pronounced for migrants, especially among girls. Compared to 2014, migrant girls were less likely to report high levels of overall health, life satisfaction, and self‐confidence in 2022. Migrant girls were also more likely to self‐report feeling sad or hopeless in the past year and more likely to report a higher frequency of health complaints in 2022 than in 2014. Such findings are of great concern and consistent with findings from research on adolescent mental health that girls fare worse on mental health and well‐being than boys [9].

In addition to psychological measures, our study used the health complaint measure which incorporates psychological and physical health indicators [38]. Migrant girls were less likely to report a high frequency of health complaints compared with nonmigrant girls. Another One possible explanation is that migrant youth may under‐report health complaints if they perceive that they are under pressure to succeed in their own and their family's eyes [16]. Other evidence suggests that migrant adolescents appear to exhibit greater resilience resources compared to nonmigrants despite experiencing more traumatic events [8]. Migrants show more resilience when faced with adversity compared with their counterparts [17, 44, 45]. This is well explained by Motti‐Stefanidi and colleagues [44] where they provide a multi‐level, systems‐approach framework. Resilience is a two‐part concept and constitutes (i) people who have faced stress or adversity and (ii) their well adaptation to this occurrence. Migrants report higher resilience since they faced adverse conditions in their migration and relocation [46]. The authors found that these adverse conditions positively influence adjustment in other domains [46]. These findings are corroborated by other findings. In a study across six countries (Australia, New Zealand, UK, China, South Africa, and Canada), migrant adolescents reported higher numbers of traumatic events compared with nonmigrant adolescents [47]. Also in line with Motti‐Stefanidi and colleagues' [44] framework, Gatt et al. [8] also found that migrants showed greater resilience towards traumatic events.

To help contextualize the trends analysis results, we convened a youth advisory group comprised of migrant youth from across Canada. Advisory members engaged in a lively discussion on the results of the trends analysis as well as factors influencing youth mental health from 2014 to 2022. Group members discussed differences in how health is viewed and talked about across cultures. When discussing the reasons for low health complaints among migrant youth with respect to nonmigrant youth, advisory group members observed that data may reflect migrant boys' tendency to underreport mental health concerns out of fear of being stigmatized, a fear rooted in cultural views about mental illness. One boy member stated his view of migrant boys wanting to be seen as “not crazy, I'm fine.” Members advised that migrant girls may be reporting fewer health complaints due to cultural stigma, or preference for traditional remedies.

Youth identified the COVID‐19 pandemic as a significant potential contributor to the decline in self‐rated overall health in 2018 and 2022 compared to 2014. Interestingly, group members also shared that support for international students and an increased frequency in community discussions about mental health during the COVID‐19 pandemic may have increased reporting of mental health concerns post‐2020. The group identified the growing importance of social media apps in the lives of many migrant youth as having a positive effect on their sense of cultural pride, potentially bolstering their self‐confidence. Young people expressed their view that a cultural shift has been occurring in the past several years from cultural appropriation to cultural appreciation. Migrant youth said they feel more comfortable embracing their cultural attire and traditions, which had once been mocked. Members shared that many migrant youth are now embracing their cultural identities rather than being pressured to assimilate, reflecting on a 2020s shift to cultural pride. Important contributors to this shift identified by group members included the Black Lives Matter movement, which may have empowered youth to speak up about any discrimination they experience. Some youth noted that people of color and migrant girls have now become “the blueprint,” making them more confident, vocal, and proud. At the same time, group members stated their view that the algorithmic nature of social media may facilitate social and physical comparison, particularly among girls, which could negatively affect their self‐esteem.

### 3.1 Implications for School Health Policy, Practice, and Equity

Canadian adolescents have faced significant challenges in the years leading up to 2022. The COVID‐19 pandemic may have had adverse effects on young people's mental health, a pandemic which may have disproportionately affected migrant youth who are already facing barriers to accessing mental health services [48]. This study provides an evidentiary basis for key stakeholders at provincial and federal levels to invest resources into programs that target adolescent mental health, particularly for migrant girls and young people who are facing financial difficulties. We recommend future studies explore serious mental health symptoms given the limitation of not asking participants about serious mental illnesses or conditions.

Given the significant barriers young migrants face in accessing mental health services [11, 12], interventions which are culturally safe and relevant, low‐ or no‐cost, and confidential are needed. Respectful language, comfort, and appreciation for cultural nuances are all highly important considerations in the provision of effective services to address migrant adolescents'' mental health needs. Schools are an ideal location for the provision of mental health programs because they are places where youth frequently are, potentially enhancing the possibility of migrant youth to receive support [49]. Ideal mental health services would have easily accessible avenues for school‐based systems to collaborate with community‐based mental health care to provide a coordinated response for migrant youth in Canada [50]. Low income has been identified as a challenge and burden for migrant youth and their families [11]. We suggest that community coordination to address poverty could have great returns.

### 3.2 Limitations

The wording of items in the HBSC survey was limited to whether a young person was born in Canada and length of time in the country; therefore, no nuance for the permanence of a respondent's status (e.g., student, citizen, etc.) could be determined. For example, there were no items to differentiate type of status (e.g., temporary or permanent). There is some evidence to suggest that health outcomes among migrants can vary depending on status and length of stay in the host country [38, 39, 40]. Information about an individual's experience, or potential contributors to their migration status prior to arriving in Canada, their status in Canada, and future in Canada are unavailable and can be explored in future research.

Since the focus of this measure is on well‐being rather than diagnosable mental illness, the data do not provide measures of diagnostic categories of mental health or more severe aspects of mental health like suicidality, self‐harm, or diagnostic measures of depression. Future iterations of the study should include the ability to explore serious mental health symptoms.

## 4 Conclusions

This study identifies early stages of erosion to well‐being at the population level and trends over time regarding mental health outcomes. The sample was weighted, population‐based, and stratified across Canada's public schools. The methodology allowed us to make inferences about whether gaps in mental health outcomes are widening, narrowing, or consistent across years between migrant and nonmigrant youth. Our findings demonstrate a decline in the prevalence of positive mental health outcomes and an increase in the prevalence of negative mental health outcomes over time, particularly for girls but especially for migrant girls. At the same time, migrant youth exhibited fewer recurring health complaints and migrant girls reported greater self‐confidence than their nonmigrant peers. We discussed results in terms of resilience and recommend investment in school‐based mental health supports for migrant youth, particularly girls, that are affordable, culturally safe/relevant, and confidential with accessible channels for school‐based systems to collaborate with community‐based supports.

## Funding

This work was supported by Canada First Research Excellence Fund (AWD‐025998).

## Ethics Statement

This study was approved by ethics boards at the Public Health Agency of Canada (REB‐2013–0022) and Queen's University's Research Ethics Board (6027003).

## Consent

The authors have nothing to report.

## Conflicts of Interest

The authors declare no conflicts of interest.

## Data Availability

The data that support the findings of this study are available from The Health Behaviour in School‐aged Children study. Restrictions apply to the availability of these data, which were used under license for this study. Data are available from the author(s) with the permission of The Health Behaviour in School‐aged Children study.
